# The IL23R R381Q Gene Variant Protects against Immune-Mediated Diseases by Impairing IL-23-Induced Th17 Effector Response in Humans

**DOI:** 10.1371/journal.pone.0017160

**Published:** 2011-02-22

**Authors:** Paola Di Meglio, Antonella Di Cesare, Ute Laggner, Chung-Ching Chu, Luca Napolitano, Federica Villanova, Isabella Tosi, Francesca Capon, Richard C. Trembath, Ketty Peris, Frank O. Nestle

**Affiliations:** 1 St. John's Institute of Dermatology, King's College London and NIHR Biomedical Research Centre, London, United Kingdom; 2 Department of Medical and Molecular Genetics, King's College London and NIHR Biomedical Research Centre, London, United Kingdom; 3 Department of Dermatology, University of L'Aquila, L'Aquila, Italy; La Jolla Institute of Allergy and Immunology, United States of America

## Abstract

IL-23 and Th17 cells are key players in tissue immunosurveillance and are implicated in human immune-mediated diseases. Genome-wide association studies have shown that the *IL23R* R381Q gene variant protects against psoriasis, Crohn's disease and ankylosing spondylitis. We investigated the immunological consequences of the protective *IL23R* R381Q gene variant in healthy donors. The *IL23R* R381Q gene variant had no major effect on Th17 cell differentiation as the frequency of circulating Th17 cells was similar in carriers of the *IL23R* protective (A) and common (G) allele. Accordingly, Th17 cells generated from A and G donors produced similar amounts of Th17 cytokines. However, IL-23-mediated Th17 cell effector function was impaired, as Th17 cells from A allele carriers had significantly reduced IL-23-induced IL-17A production and STAT3 phosphorylation compared to G allele carriers. Our functional analysis of a human disease-associated gene variant demonstrates that *IL23R* R381Q exerts its protective effects through selective attenuation of IL-23-induced Th17 cell effector function without interfering with Th17 differentiation, and highlights its importance in the protection against IL-23-induced tissue pathologies.

## Introduction

Increasing understanding of the mechanisms underpinning immune-mediated inflammatory diseases such as psoriasis, Crohn's disease (CD) and ankylosing spondylitis (AS) has implicated a pivotal role for the IL-23/Th17 cells axis in their pathogenesis [1], [2], [3], [4].

IL-23 consists of the unique IL-23p19 subunit coupled with the common IL-12p40 subunit (shared with IL-12) [5]. It is mainly produced by activated myeloid cells, as well as epithelial and endothelial cells, and signals through its heterodimeric IL-23R complex [6]. This complex consists of the IL-23R subunit paired with the IL-12Rβ1 subunit shared with the IL-12R complex. Binding of IL-23 to IL-23R complex leads to STAT3 phosphorylation, and IL-23-dependent gene expression.

IL-23 is a key pro-inflammatory cytokine driving autoimmunity in animal models and human diseases. In mice, lack of IL-23 makes them resistant to experimental models of arthritis and multiple sclerosis (MS) [7], [8]. We and others have shown that selectively targeting IL-23 prevents auto-immune inflammation in experimental models of MS [9], inflammatory bowel disease [10], [11] and in a clinically relevant psoriasis model [12]. In humans, IL-23 is over-expressed in clinical samples of psoriasis [13], CD [14] and AS [15] and an anti-IL-12/IL-23p40 mAb is efficacious in treating psoriasis and CD [16], [17]. IL-23 plays a critical role in Th17 response and production of the lineage-defining cytokine IL-17A [18], [19], [20]. Human Th17 cells express the master transcription factor RORC and the surface markers CCR6, IL-23R and CD161 and they differentiate in the presence of TGF-β1 and at least one pro-inflammatory cytokine such as IL-1β, IL-6, IL-21 and IL-23 [21], [22]. In addition to IL-17A, IL-17F and IL-26, Th17 cells produce cytokines shared with other Th cell subsets such as IL-22 and IFN-γ [23], [24]. Th17 cells drive autoimmunity in experimental models [7], [25] and have been identified in clinical samples of psoriasis [26] and CD [27]. Although not required for early stages of Th17 development, as naïve T cells express little or no IL-23R [6], IL-23/IL-23R signalling plays a critical role in favouring terminal differentiation, maintenance and pathogenicity of effector Th17 cells [28], with IL-23 driving local Th17 effector response. In animal models of intestinal inflammation IL-23 acts as a key tissue-specific effector cytokine amplifying the inflammatory response [10], [11], [29]. Intradermal injection of IL-23 results in skin inflammation in mice [30] and delivery of exogenous IL-23 in IL-23p19 KO mice restores susceptibility to autoimmune diseases [8], [28].

Strong evidence for the importance of the IL-23/Th17 axis in immune-mediated diseases has emerged from genetics studies. One of the most robust genetic findings is the association of a variant in the *IL23R* gene with CD [31], psoriasis [32], [33] and AS [34]. We and others have found that the frequency of a single-nucleotide polymorphism (SNP) in the *IL23R* is significantly higher among healthy controls than in patients, suggesting a protective effect of the rare allele from immune-mediated chronic inflammation. The associated SNP, consisting in a guanine (G) to adenine (A) substitution at DNA level, results in an arginine (R) to glutamine (Q) substitution in position 381 (R381Q) within the cytoplasmic domain of the IL-23R. Although this genetic association has been replicated, the functional consequences of carrying the protective gene variant are yet to be determined. One possibility is that the *IL23R* R381Q SNP protects from multiple immune-mediated diseases by impairing IL-23-mediated Th17 responses.

In this study we provide a comprehensive functional characterization of the protective *IL23R* R381Q gene variant in healthy donors. We found that the *IL23R* R381Q SNP had no major effect on Th17 cell differentiation; however, IL-23-induced Th17 cell effector function was impaired in protective allele carriers resulting in significantly reduced IL-17A production and STAT3 phosphorylation. These results support a critical role for the IL-23/IL-23R signaling in generating pathogenic Th17 response.

## Materials and Methods

### Ethics Statement

This study was approved by the institutional review board of Guy's Hospital (Guy's Research Ethics Committee, Ethics Committee Code: 06/Q0704/18) and conducted in accordance with the Helsinki Declaration, with informed written consent obtained from each volunteer.

### Healthy volunteers and Genotyping

Healthy individuals of Western European descent were selected on the exclusion of personal or family history of immune-mediated disorders, and genotyped for the *IL23R* R381Q variant. The typing of 176 individuals resulted in no subjects homozygous for the rare A allele (AA), 30 heterozygous A (A group) and 146 homozygous for the common G allele (G group). We used 41 donors (14 males, 27 females; mean age 34 years, range 23–65 years) to perform our functional studies: 19 A and 22 G individuals. Genomic DNA was extracted from peripheral blood by using a commercially available kit (Macherey Nagel, Düren, Germany), according to manufacturers' instructions. *IL23R* R381Q SNP was genotyped as previously described [33].

### CD4+ T cells and PBMCs isolation

CD4+ T cells (purity over 98%) were isolated from peripheral blood by incubation with Rosette Sep Human CD4+T cells enrichment cocktail (StemCells Technologies, Grenoble, France) followed by centrifugation on a density gradient (Lymphoprep, PAA, Pasching, Austria). PBMCs were purified by centrifugation through Lymphoprep.

### Flow Cytometry

For cellular surface staining the following antibodies and secondary reagents were used in different combinations: biotinylated goat anti-human IL-23R (BAF1400, R&D System, Minneapolis, MN) [12], [19], [35], Streptavidin-APC (BD Bioscience, San Jose, CA), CD3-FITC (eBioscience, San Diego, CA), CD4 PE-Texas Red (Invitrogen, Carlsbad, CA), CD45RO-FITC (Dako, Glostrup, Denmark), CD45RO-Pacific Blue (BioLegend, San Diego, CA), CCR6-PE (BD Bioscience), CD45RA-PE (Invitrogen), CD45RA-PE-Cy7 (eBioscience), plus matched isotypes as controls. Cells were acquired on a BD FACSAria II (BD Bioscience). Analysis of FACS data was performed by FlowJo (TreeStar, Ashland,OR) software.

### Intracellular cytokines staining

PBMCs were activated with PMA (50 ng/ml, Sigma, St.Louis, MO) plus ionomycin (250 ng/ml, Calbiochem, Darmstadt, Germany) in the presence of monensin (3 µM, Sigma) for 5 hours. Cells were subsequently stained for surface markers, fixed and permeabilized in Fix Buffer and Permeabilization Buffer (eBioscience) according to manufacturers' instructions and stained with IL-17A-PE (eBioscience) or matching isotype control.

### Generation of Th17 cells from naïve CD4+ T cells

Highly purified CD4^+^CD45RA^+^CD45RO^−^ naïve T cells were obtained by negative selection of CD4^+^ T cells using magnetic beads (Dynabeads Pan Mouse IgG, Invitrogen) and the following purified antibodies against unwanted cells: CD8 (Invitrogen), TCRγ/δ (BD Bioscience), CD19 (Diaclone, Besançon, France), CD16 (Diaclone), CD14 (Diaclone), CD33 (Invitrogen), CD56 (Diaclone), CD45RO (Invitrogen).

Purity of CD4^+^CD45RA^+^CD45RO^−^ naïve T cells was checked by FACS and considered acceptable if over 95%. CD4^+^CD45RA^+^CD45RO^−^ T cells were then polarized to Th17 phenotype according to a published protocol [19] with some modifications. Of note, TGF-β in our cell culture came from human AB serum and upon checking by ELISA, using a commercially available TGF-β ELISA kit (R&D Systems) according to manufacturers' instructions, resulted equal to 0.4±0.1 ng/ml, within the optimal range (0.1–1 ng/ml) reported for Th17 polarization [20]. Briefly, cells were cultured at density of 5×10^5^ cells/ml in U-bottomed 96-well plates in RPMI medium containing 10% human AB serum (Biowhittaker, Walkersville, MD) (10% RPMI-hAB) along with anti-CD3/CD28 coated beads (1 beads per cell, Dynabeads CD3/CD28 T cell Expander, Invitrogen) and recombinant human IL-1β (50 ng/ml, Peprotech, Rocky Hill, NJ), where required, for 6 days. On d6 cells were counted and expanded for additional 7 d using IL-2 (100 UI/ml, R&D Systems) and IL-1β in the presence or absence of IL-23 (50 ng/ml, R&D Systems). On d13 cells were counted, washed and used for flow cytometry analysis, RNA extraction or further re-stimulated as described below.

### Th17 cell activation

To determine cytokine mRNA levels, IL-1β/IL-23 or IL-1β-polarized cells on d13 of culture were left un-stimulated or stimulated with IL-23 (10 ng/ml) for 24 h. Cells were stored in TRIzol reagent (Invitrogen) at −80°C until further use.

Cells from each culture condition were either unstimulated or stimulated with PMA (5 ng/ml) plus ionomycin (3 µg/ml), or IL-23 (10 ng/ml), for 48 h to determine cytokine production. Cell–free supernatant was collected and stored at −80°C until further use.

### RNA extraction and quantitative RT-PCR (qRT-PCR)

Total RNA was obtained using TRIzol according to the manufacturers' instructions and reverse transcribed into cDNA. RORC, IL-23R, CCR6, IL-17A, IL-17F, IL-22 and IL-26 expression was assessed by multiplex real-time quantitative RT-PCR using Taqman assays (Applied Biosystems, Carlsbad, CA) according to the manufacturers' instructions. For each sample, mRNA abundance was normalized to the amount of human GAPDH. Data analysis was performed using the ΔΔCt method: results are expressed either as fold change or as relative mRNA levels in arbitrary units.

### Determination of IL-17A, IFN-γ and IL-22 levels

IL-17A and IFN-γ cytokine levels in the supernatants were assayed using the Fluorokine MAP Human Base Kit A (R&D Systems) and acquired on a Luminex 100 flow-based sorting and detection analyzer (Luminex Corporation, Austin, TX).

IL-22 cytokine level was assayed using a commercially available IL-22 ELISA kit (R&D Systems) according to manufacturers' instructions.

### pSTAT-3 intracellular staining

For determination of pSTAT-3 IL-1β-polarized cells were rested for 24 h and either left un-stimulated or stimulated with IL-23 (10 ng/ml) for 15 min. Cells were fixed, permeabilized and stained with pSTAT3 (Tyr 705) (Cell Signaling, Danvers, MA) and secondary goat anti-rabbit-Alexa 488 (Invitrogen), according to the manufacturers' instructions.

### Statistical analysis

For flow cytometry experiments cell percentages and MFI values obtained for each donor belonging to the two genetic groups were assessed for normal Gaussian distribution with D'Agostino & Pearson omnibus normality test and then analyzed by unpaired two-tailed t test by using Prism version 4.0 (GraphPad Software, La Jolla, CA). For cytokine secretion experiments cell supernatants were assayed in duplicates, mean ± SEM was calculated, results were assessed for normal Gaussian distribution and then analyzed by Mann-Whitney test. For mRNA expression analysis qRT-PCR was performed in triplicates, mean ± SD was calculated, results were assessed for normal Gaussian distribution, and then analyzed by Mann-Whitney, unpaired two-tailed *t* or Wilcoxon signed rank test, as appropriate. Values of P<0.05 were considered significant.

## Results

### Study of circulating Th17 cells in IL23R R381Q gene variant carriers

As a first step to study the functional consequences of the *IL23R* R381Q gene variant we chose an appropriate study population. We studied a population of healthy donors rather than patients, due to the possible presence of more confounding gene variants in patients. Out of 176 individuals typed for the *IL23R* R381Q SNP, 30 individuals were heterozygous for the protective A allele (A group) and 146 individuals homozygous for the common G allele (G group). No subjects were homozygous for the very rare A allele (AA), in keeping with its genotype frequency (less than 1% AA homozygous in individuals of Western European descent, HapMap project, public release #27). We ultimately used 19 A and 22 G individuals for functional studies.

Given the clear link between IL-23 and Th17 cells, we hypothesized that the *IL23R* R381Q SNP could affect IL-23/IL-23R signalling, impairing Th17 responses.

**Figure 1:**
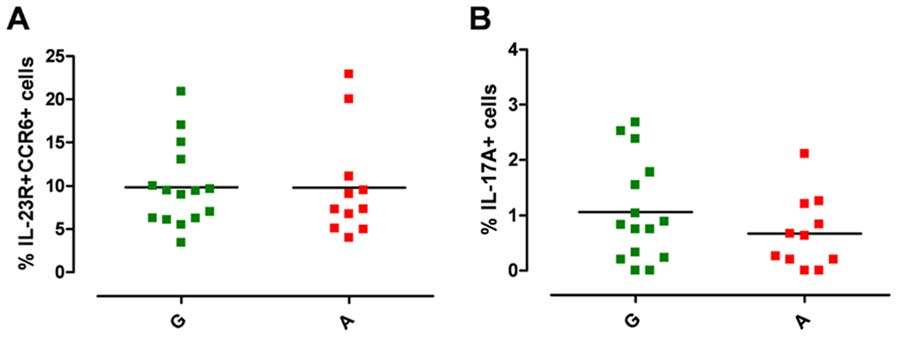
*IL23R* R381Q gene variant and circulating Th17 cells. PBMCs from normal G (green squares) or protected A (red squares) donors were stained for Th17 cell surface markers and for IL-17A production. (**A**) Percentage of IL-23R^+^CCR6^+^ Th17 cells and (**B**) of IL-17A^+^ cells, within memory Th cells. Each symbol corresponds to one individual, horizontal bars represent means. Unpaired *t* test was performed yielding P values>0.05 for both panels.

To test our hypothesis we first studied freshly isolated PBMCs in the absence of cell expansion or manipulation and investigated whether there was any difference in circulating Th17 cells, between the A and G group. Human Th17 cells were defined by surface co-expression of IL-23R and CCR6 and by the production of IL-17A in response to polyclonal stimulation, as, in keeping with a published study [35], IL-23 stimulation was unable to consistently induce a detectable IL-17A response in freshly isolated memory Th cells from healthy donors (data not shown). As previously shown [36], we observed individual variation within our cohort of healthy donors, in both the percentage of circulating Th17 cells and of IL-17A^+^ cells (Fig. 1A, B). Percentage of IL-23R^+^CCR6^+^ Th17 cells within memory Th cells ranged from 3.42 to 22.90 (Fig. 1A). There was no significant difference neither in the percentage of Th17 cells (Fig. 1A, Fig. S1A), nor in IL-23R mRNA expression (Fig. S1B) or in the percentage of IL-23R^+^ cells (Fig. S1C, D) and IL-23R Median Fluorescence Intensity (MFI) (Fig. S1E, F) in purified Th cells between the A *vs* the G group. The percentage of IL-17A^+^ cells within the memory Th cells ranged from 0.01 to 2.68 (Fig. 1B, Fig. S1G). While there was a slight reduction in percentage of IL-17A^+^ cells in the protected A group, this did not reach statistical significance. Such subtle differences, although not conclusively able to exclude impairment in Th17 cells generation in protective A allele carriers, leave open the possibility that environmental and/or immunological factors, rather than the *IL23R* R381Q SNP, might have impacted on Th17 cell development and function in our cohort. A similar scenario, described also by others [36], highlights that the degree of inter-individual variability in Th repertoire composition provides a challenge when assessing functional consequences of gene variants. To overcome such inter-individual variability, we established a cell culture system for the generation of *in vitro* polarized Th17 cells which enabled us to pinpoint subtle functional impacts of the *IL23R* SNP and to discriminate between IL-23 effect in Th17 differentiation and effector functions.

### Effect of the IL-23R R381Q gene variant on IL-23-induced Th17 cell differentiation

**Figure 2:**
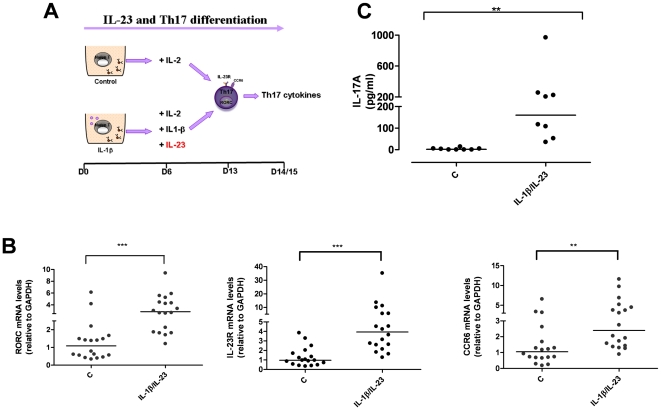
IL-1β/IL-23-polarized cells express Th17 markers and produce IL-17A. (**A**) Naive CD4^+^ T cells were cultured for 6 d in the presence of anti-CD3/CD28 coated beads, with or without IL-1β. On d6 cells were counted and IL-2 added to each condition, together with IL-1β and IL-23, where required. IL-1β/IL-23-polarized Th17 cells were collected after 13 d of culture for phenotypic analysis or rested and assayed for Th17 cytokine on d14/15. (**B**) mRNA expression levels of RORC, IL-23R and CCR6 on d13 were significantly increased in IL-1β/Il-23-polarized cells. (**C**) IL-17A production on d15 was significantly enhanced in IL-1β/Il-23-polarized cells. Results are representative of 5 experiments. Each symbol corresponds to one individual, horizontal bars represent medians. Wilcoxon signed rank test was performed. ** P<0.01, ***P<0.001.

Although dispensable in the first stage of human Th17 differentiation, IL-23 is required to drive terminal differentiation and maintenance of already committed Th17 cells [21]. To investigate whether the *IL23R* R381Q SNP has an effect on IL-23-induced Th17 differentiation, we generated Th17 cells with IL-1β, in the presence of IL-23 added at d6 of culture during cell differentiation (Fig. 2A). On d13/15 the cells showed phenotypic and functional characteristics of fully differentiated effector Th17 cells. IL-1β/IL-23-polarized cells expressed higher mRNA levels of RORC (P<0.001), IL-23R (P<0.001) and CCR6 (P<0.01), (Fig. 2B) and produced significantly higher amounts of IL-17A cytokine (P<0.01) and IL-17F and IL-26 mRNA (Fig. 2C and not shown) compared to un-polarized control cells.

**Figure 3:**
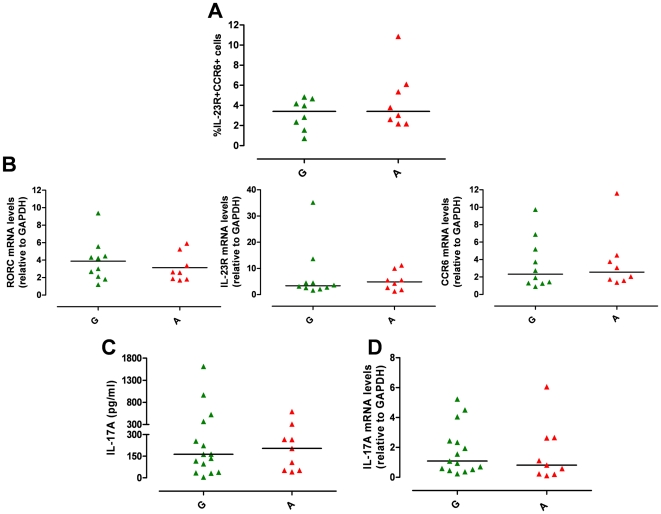
*IL23R* R381Q gene variant does not affect Th17 differentiation. IL-1β/IL-23-polarized Th17 cells were used for phenotypic analysis (on d13) or assayed for IL-17A mRNA expression (on d14) or secretion (on d15). (**A**) Percentage of CCR6^+^IL-23R^+^ IL-1β/IL-23-polarized Th17 cells did not significantly differ between G (green triangles) and A group (red triangles). (**B**) mRNA expression levels of RORC, IL-23R and CCR6 did not differ between G and A group. (**C**) IL-17A production and (**D**) IL-17A mRNA expression did not differ between G and A group. Each symbol represents one individual, horizontal bars represent medians. Mann Whitney test was performed yielding P values>0.05 for all panels.

There was no difference between A and G group in the percentage of IL-23R^+^CCR6^+^ Th17 cells (P>0.05, Fig. 3A) and in mRNA levels of RORC, IL-23R and CCR6 (P>0.05, Fig. 3B). IL-17A production at protein (IL-17A: G group median = 163 pg/ml, from 5 to 1612 pg/ml, n = 15 *vs* A group median = 204 pg/ml, from 42 to 591, n = 9; P>0.05, Fig. 3C) and mRNA level (IL-17A mRNA levels: G group median = 1.09, from 0.23 to 5.23, n = 15 *vs* A group median = 0.81, from 0.10 to 6.06, n = 9; P>0.05, Fig. 3D) did not differ between the two groups. Similarly there was no difference in IL-17F and IL-26 mRNA (Fig. S2A, B) and in IL-22 production at both protein and mRNA levels (Fig. S2C, D) between A and G group. Temporal analysis of IL-23R expression showed no difference in the percentage of IL-23R^+^ cells (Fig. S2E) and in IL-23R MFI (Fig. S2F) between A and G group.

These results obtained with *in vitro* polarized Th17 cells in the presence of IL-23 suggest that the *IL23R* R381Q SNP does not affect human Th17 differentiation and are consistent with those obtained with freshly isolated circulating Th17 cells.

### IL-23R R381Q gene variant impairs Th17 effector function

**Figure 4:**
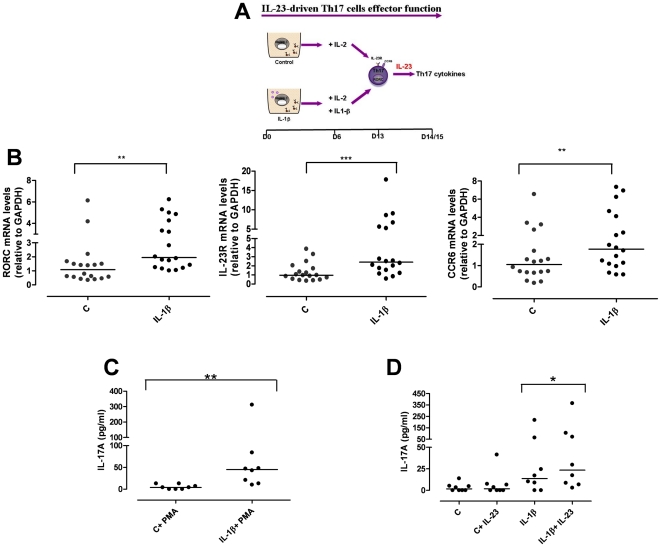
IL-1β-polarized cells express Th17 markers and produce IL-17A in response to IL-23. (**A**) Naïve CD4^+^ T cells were cultured for 6 d in the presence of anti-CD3/CD28 coated-beads, with or without IL-1β. On d6 cells were counted and IL-2 added to each condition, together with IL-1β, where required. IL-1β-polarized Th17 cells were collected after 13 d of culture for phenotypic analysis or assayed for Th17 cytokine on d14/15. (**B**) mRNA expression levels of RORC, IL-23R and CCR6 on d13 were significantly increased in IL-1β-polarized cells. (**C**) IL-17A production in response to PMA/Ionomycin (PMA) was significantly enhanced in IL-1β-polarized cells. (**D**) IL-1β-polarized cells produced higher level of IL-17A in response to IL-23 than control cells. Results are representative of 5 experiments. Each symbol corresponds to one individual, horizontal bars represent medians. Wilcoxon signed rank test was performed. *P<0.05, **P<0.01, ***P<0.001.

Accumulating evidence suggests a pathogenic role for IL-23 in inflamed epithelial tissues. We wondered if the *IL23R* R381Q SNP would impair IL-23-induced Th17 effector function. To mimic Th17 cell response in inflamed tissue we studied the effect of short-term IL-23 stimulation on already committed Th17 cells at d13 of culture (Fig. 4A). Phenotypic characterization of IL-1β-polarized Th17 cells showed a higher mRNA expression of RORC (P<0.01), IL-23R (P<0.001) and CCR6 (P<0.01) compared to un-polarized control cells (Fig. 4B). When IL-1β-polarized cells were polyclonally stimulated for a further 48 h they produced statistically significant more IL-17A (P<0.01), IL-22, and IFN-γ cytokines as well as IL-17F and IL-26 mRNA (Fig. 4C and data not shown) compared to non-polarized control cells.

We next assessed whether Th17 cells would respond to 48 h IL-23-stimulation by producing IL-17A and other Th17 cytokines. As shown in Fig. 4D, IL-17A production was significantly (P<0.05) increased in IL-23-stimulated IL-1β-polarized Th17 cells compared to unstimulated cells. In contrast, un-polarized control cells did not respond to IL-23. IL-1β-polarized cells also expressed IL-17F and IL-26 mRNA, as well as IL-22 and IFN-γ in response to IL-23 (data not shown).

**Figure 5:**
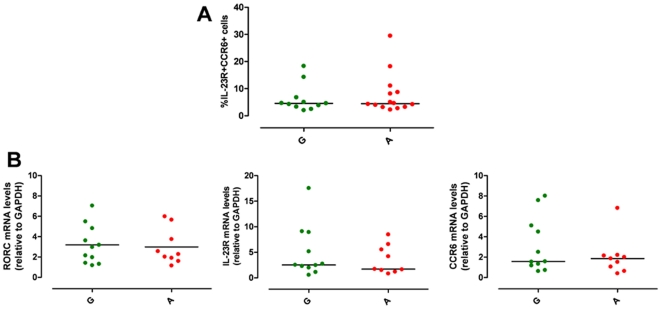
*IL23R* R381Q gene variant and IL-1β-polarized Th17 cells. IL-1β-polarized Th17 cells were used for phenotypic analysis on d13. (**A**) Percentage of CCR6^+^IL-23R^+^ IL-1β-polarized cells did not significantly differ between G (green triangles) and A group (red triangles). (**B**) mRNA expression levels of RORC, IL-23R and CCR6 did not differ between G and A group. Each symbol represents one individual donor; horizontal bars represent medians (A) or means (B). Mann Whitney test (A) or unpaired *t* test (B) were performed yielding P values >0.05 for all panels.

Immunophenotypic analysis of IL-1β-polarized Th17 cells revealed that the percentage of d13 CCR6^+^IL-23R^+^ Th17 cells did not differ significantly between the two genetic groups (A *vs* G, P>0.05) (Fig. 5A). This was also true for the relative mRNA levels of RORC, IL-23R and CCR6 (A *vs* G, P>0.05) (Fig. 5B). Similarly, temporal analysis of IL-23R expression showed no difference in the percentage of IL-23R^+^ cells (Fig. S3A) and IL-23R MFI (Fig. S3B) between A and G groups. The two genetic groups showed also a similar up-regulation of IL-23R mRNA in response to IL-23-stimulation for 48 h (Fig. S3C). Thus, IL-1β-polarized Th17 cells represented a suitable tool to study the impact of the *IL23R* R381Q SNP in IL-23-induced Th17 effector response, as Th17 cells from A and G group were similar not only numerically and phenotypically but also in their capacity to respond to IL23 as they had transcriptionally up-regulated the IL-23R at the same level.

**Figure 6:**
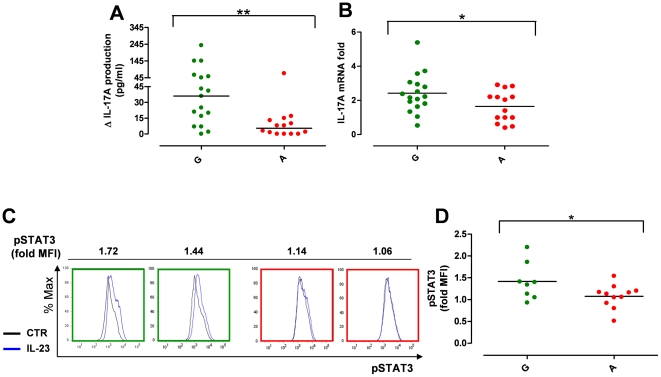
*IL23R* R381Q gene variant reduces IL-23-induced IL-17A production and STAT3 phosphorylation. IL-1β-polarized Th17 cells collected on d13 were stimulated with IL-23. (**A**) IL-17A production in response to 48 h IL-23 stimulation was significantly reduced in IL-1β-polarized Th17 cells from A (red dots) compared to G (green dots) group. Data are expressed as net IL-17A production in response to IL-23. (**B**) IL-17A mRNA expression in response to 24 h IL-23 stimulation was significantly reduced in IL-1β-polarized Th17 cells from A compared to G group. Data are expressed as fold increase compared to unstimulated cells. (**C**) Representative flow cytometry histograms from G (green frame) and A (red frame) donors showing STAT-3 phosphorylation in response to 15 min IL-23-stimulation (blue line) as compared to unstimulated control cells (black line) and expressed as fold Median Fluorescence Intensity (MFI). (**D**) STAT3 phosphorylation in response to IL-23-stimulation was reduced in IL-1β-polarized cells from A compared to G group. Each symbol represents one individual donor; horizontal bars represent medians (A) or means (B, D). Mann-Whitney (A) or unpaired *t* test (B, D) was performed, *P<0.05 **P<0.01.

When IL-1β-polarized Th17 cells were stimulated with IL-23 for 48 h we detected a statistically significant reduction in IL-23-induced IL-17A net production in protective A allele carriers compared to the group carrying only G alleles (G group median = 36.0 pg/ml, from 0 to 238 pg/ml, n = 17 *vs* A group median = 5.5 pg/ml, from 0 to 71 pg/ml, n = 14; P<0.01) (Fig. 6A). We also found a significant reduction of IL-17A mRNA in response to IL-23 in the Th17 cells from A versus G group (IL-17A mRNA fold increase G group mean = 2.43, from 0.53 to 5.40, n = 17 *vs* A group mean = 1.65, from 0.41 to 2.92, n = 14; P<0.05) (Fig. 6B). These data suggest that the R381Q gene variant negatively affects IL-23 signal transduction and ultimately *IL17A* transcription. We tested the impact of the *IL23R* R381Q SNP on IL-23 signalling, focusing on STAT3 phosphorylation (pSTAT3). To detect and quantify small differences in pSTAT3 at single cell level [37] we performed phospho-flow cytometry analysis, providing us with both a higher sensitivity and specificity than conventional western blot analysis (data not shown). We found that the magnitude of IL-23-induced pSTAT3 in Th17 cells (expressed as fold MFI relative to unstimulated cells [37]) was significantly reduced in the A *vs* G group (Fig. 6C, D). This reduction was specific for IL-23R signalling as there was no variation in pSTAT3 between the A and G group when Th17 cells were stimulated with the major pSTAT3-inducing cytokine IL-6 (Fig. S3D, E). IL-23 stimulation of Th17 cells from group A and G did not show a statistically significant difference in cytokine production and mRNA expression of pro-inflammatory cytokines (IL-17F, IL-22, IL-26, IFN-γ) (Fig. S3F-I).

Thus, we detected a significantly reduced production of IL-17A as well as impaired STAT3 phosphorylation in Th17 effector cells from carriers of the protective *IL23R* gene variant.

## Discussion

In this study we provide the first functional characterization of the *IL23R* R381Q SNP showing an impaired IL-23-induced Th17 response in carriers of the A protective allele. Using PBMCs from healthy donors we detected no major difference in the number and activity of circulating Th17 cells between G and A allele carriers. In keeping with these results, Th17 cells generated *in vitro* from naïve T cells in the presence of IL-23 consistently showed a similar phenotype and functional activity in both G and A group. However, effector Th17 cells from individuals carrying the protective *IL23R* SNP produced significantly less IL-17A in response to IL-23 and had impaired IL-23 signalling at the level of pSTAT3.

The IL-23/Th17 axis has received considerable interest in the past few years, as it is key both in protective immunity against infections and pathogenic in some autoimmune-type diseases, such as psoriasis, CD and AS. Tissue-derived IL-23 can drive pathogenic Th17 effector responses leading to overwhelming inflammation and autoimmunity. Genetic studies have shown that the less common A allele of the *IL23R* confers approximately threefold protection against developing CD [4] and twofold protection against psoriasis [38] and AS [34].

In this study we used Caucasian healthy donors with no personal or family history of psoriasis, CD or AS. We chose not to investigate the effect of the *IL23R* R381Q SNP in patients as we assumed that the protective effect of the *IL23R* variant might have been partially overcome by other genetic or environmental factors that contribute to disease pathogenesis. Due to the low frequency of the genetic variant of interest (<1% AA homozygous in individuals of Western European descent), which might be based on a negative evolutionary selection of the A allele to preserve an adequate Th17 response against infection, we only had access to individuals that were heterozygous for the *IL23R* R381Q gene variant.

**Figure 7:**
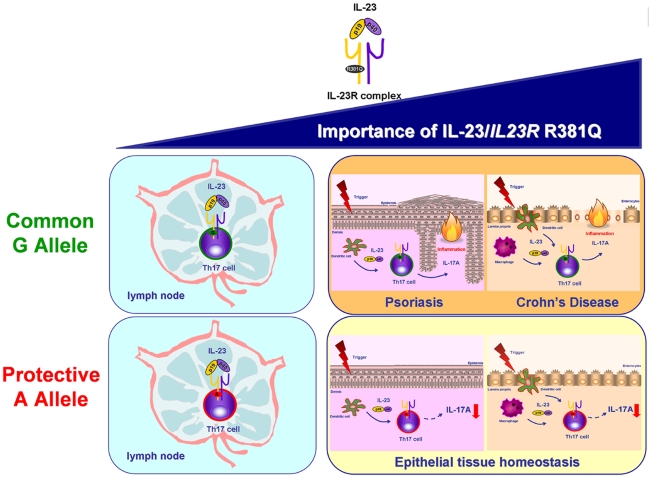
Importance of IL-23 and *IL23R* R381Q gene variant in Th17 cell immunobiology. The IL-23/IL-23R axis is believed to play a role in both Th17 differentiation in the lymph node and Th17 cell effector functions in peripheral tissues, with the latter becoming increasingly relevant, especially in the context of IL-23-induced tissue pathologies, like psoriasis and Crohn's disease. Our data about the functional consequences of carrying the protective A allele of the *IL23R* R381Q gene variant suggest that its protective effect against autoimmune disease is driven trough impairment of Th17 cell effector functions, i.e. IL-17A production, rather than Th17 differentiation.

A key finding of our study was that the protective *IL23R* SNP affects IL-23 signalling in Th17 effector cells impairing IL-17A production *without* interfering with the differentiation of Th17 cells from naïve T cells. In fact, the number and activity of both circulating Th17 cells and *in vitro* differentiated Th17 cells did not differ between G and A allele carriers. This finding supports the current concept of a major role for IL-23 in the generation of Th17 cell effector response in tissue inflammation, rather than in systemic inflammation and demonstrates the importance of IL-23 in mediating Th17 effector response in humans. Our results suggest a protective effect of the *IL23R* R381Q SNP in IL-23-mediated IL-17A-induced peripheral tissue pathology seen in chronic inflammatory diseases (Fig. 7).

IL-23 signalling impairment only affected IL-17A production and not the other cytokines produced by Th17 cells. It has been shown that cultured Th17 cells are heterogeneous in their cytokine production with some producing solely the lineage–defining cytokine IL-17A and others secreting combinations of other Th-17 related cytokines, e.g. IL-22 and IFN-γ [23], [24]. Therefore, our findings raise the possibility that the *IL23R* R381Q SNP mainly exerts its effect on cells producing only IL-17A, while preserving an adequate overall cytokine response.

By demonstrating the immunological consequences of the protective *IL23R* R381Q SNP we have provided further insight into the role of the IL-23/Th17 axis in immune-mediated diseases. Our study is one of the first to demonstrate that functional characterization of gene variants associated with human common complex diseases is feasible. The overall magnitude of a given gene variant to protect against disease might be small, but, as in the case of the *IL23R* gene variant, there are compelling scenarios contributing to disease pathogenesis. The positive outcome of our immunogenetic study suggests that parameters such as adequate study population, sufficient statistical power, relevant cell type and pertinent biological read out are critical in discovering the functional impact of disease-associated SNPs. A study using human T cell blasts transduced with the two *IL23R* variants was unable to detect any differences in IL-23-induced STAT phosphorylation and IFN-γ and IL-10 production [39]. This finding was confirmed using mouse Ba/F3 cells infected with *IL23R* gene variants (E. Oldham and R. Kastelein, personal communication). In our study we used a more physiological model system using primary human cells from typed individuals without the need for genetic manipulation. In addition, we specifically looked at the effect of *IL23R* R381Q SNP in Th17 cells, using IL-17A as read out for Th17 cell effector function.

Finally, our results are relevant in the context of the clinical efficacy of mAbs targeting IL-12/IL-23p40 in psoriasis and CD [16], [40]. Studies are currently ongoing to investigate whether the protective *IL23R* gene variant might also contribute to a different disease phenotype in psoriasis, as it has been suggested in CD patients [41]. It is also feasible that the *IL23R* SNP could be used as a novel biomarker to predict therapeutic response and studies are currently ongoing to test this hypothesis in psoriasis. Thus, insights gleaned from “gene to function” studies could be translated into designing more efficient and cost-effective clinical trials for immune-targeted drug therapy in chronic inflammatory diseases.

## Supporting Information

Figure S1 **Effect of** ***IL23R*** **R381Q gene variant on circulating Th17 cells and IL-23R protein and mRNA expression.**
Circulating Th17 cells were analysed in PBMCs. Representative flow cytometry contour plots showing (**A**) percentage of IL23R^+^CCR6^+^ Th17 cells within CD3^+^CD4^+^CD45RO^+^ cells in G (green frame) and A (red frame) donors. Isotype controls are shown. IL-23R expression was analyzed in purified total CD4^+^ T cells by flow cytometry and qRT-PCR. (**B**) mRNA expression level of IL-23R in total CD4^+^ T cells in G (green squares) or A (red squares) donors. (**C**) Representative flow cytometry contour plots showing percentage of IL23R^+^ cells within purified CD4^+^ T cells in G and A donors. Isotype controls are shown. (**D**) Percentage of total CD4^+^ T cells expressing IL-23R in G or A donors. (**E**) Representative flow cytometry histogram showing median fluorescence intensity (MFI) as measurement of IL-23R expression in a G (green line) and an A (red line) donor. Isotype control is shown (tinted grey) (**F**) CD4^+^IL-23R^+^ T cells MFI in G (green) or A (red) healthy individuals. (**G**) Representative flow cytometry contour plots showing percentage of CD45RO^+^IL17^+^ cells within CD3^+^CD4^+^cells determined for G and A donors by intracellular cytokine staining. Isotype controls are shown Each symbol in panels B, D and F corresponds to a value obtained from an individual and horizontal bars represent means. Unpaired t test was performed yielding P values >0.05 for all panels.
(TIF)

Figure S2 **Effect of** ***IL23R*** **R381Q gene variant on Th17cell differentiation.**
IL-1β/IL-23 polarized Th17 cells were collected after 13d of culture, rested and then assayed for mRNA expression (on d14) or Th17 cytokine secretion (on d15). IL-17F (**A**) and IL-26 (**B**) mRNA and IL-22 protein secretion (**C**) and mRNA expression (**D**) in IL-1β/IL-23 polarized Th17 cells did not differ between G (green triangles) and A (red triangles) group. Time course analysis of IL-23R expression in IL-1β/IL-23 polarized Th17 cells was performed at d6 and 13 of culture by flow cytometry. Percentage of IL-23R^+^Th17 cells (**E**) and IL-23R MFI (**F**) did not differ between G (green boxes) and A (red boxes) group neither at d6 nor at d13. Box and whiskers of 5 donors per group are shown. Horizontal bars represent means (A) or medians (B, C, D, E, F). Unpaired t test (A) or Mann Whitney test (B, C, D, E, F) were performed yielding P values >0.05 for all panels.
(TIF)

Figure S3 **Effect of** ***IL23R*** **R381Q gene variant on IL-23R expression, IL-6-induced STAT-3 phosphorylation and production of pro-inflammatory cytokines.**
Time course analysis of IL-23R expression in IL-1β polarized Th17 cells was performed at d 6 and d13 of culture by flow cytometry. % of IL-23R^+^Th17 cells (**A**) and IL-23R MFI (**B**) did not differ between G (green boxes) and A (red boxes) group neither at d6 nor at d13. IL-1β-polarized Th17 cells were stimulated with IL-23 on day 13 of culture and IL-23R mRNA was measured on d14. IL-23R mRNA expression (**C**) in response to IL-23 stimulation (10 ng/ml) did not differ in IL-1β-polarized Th17 cells from A (red dots) versus G (green dots) group. mRNA were measured by qPCR and data are expressed as fold increase compared to un-stimulated cells. IL-1β-polarized Th17 cells were stimulated with IL-6 (100 ng/ml) for 15 min on d13 of culture and pSTAT-3 was measured by flow cytometry. (**D**) Representative flow cytometry histograms from two G (green frame) and two A (red frame) donors showing pSTAT-3 in response to IL-6 (pink line) as compared to unstimulated control cells (black line) and expressed as fold Median fluorescence Intensity (MFI). (**E**)Th17 cells from group G and group A did not differ in IL-6-induced pSTAT3. IL-1β-polarized Th17 cells were stimulated with IL-23 on d13 of culture and Th17 cytokine mRNA and proteins were measured on d14 or d15, respectively. IL-17F (**F**) and IL-26 (**G**) mRNA expression, as well as net IL-22 (**H**) and IFN-γ (**I**) production in response to IL-23 stimulation was not affected in IL-1β-polarized Th17 cells from A compared to G group. IL-17F and IL-26 mRNA were measured by qPCR and data are expressed as fold increase compared to unstimulated cells. For panels A, B and E box and whiskers of 4–5 donors per group are shown. In panels C, F-I each symbol corresponds to a value obtained from an individual. Horizontal bars represent medians (C, F, H, I) or means (G). Mann Whitney (C, F, H, I) or unpaired t test (G) was performed yielding P values >0.05 for all panels.
(TIF)
