# Supplementary figures and images for: The IL23R R381Q Gene Variant Protects against Immune-Mediated Diseases by Impairing IL-23-Induced Th17 Effector Response in Humans
Source: PLoS One. 2011 Feb 22;6(2):e17160. doi: 10.1371/journal.pone.0017160 (PMC3043090; doi:10.1371/journal.pone.0017160)

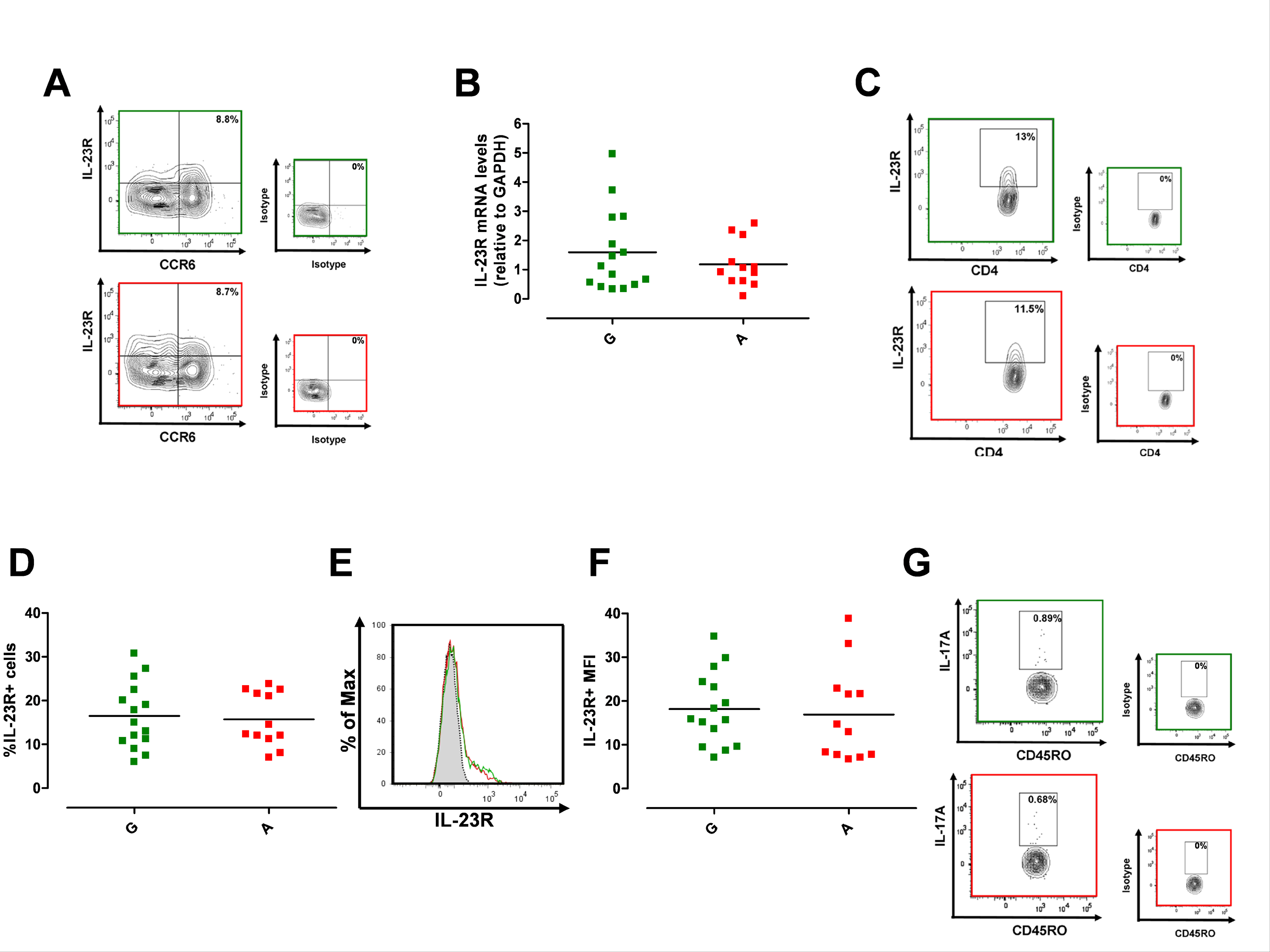

Supplement: Figure S1 — Effect of IL23R R381Q gene variant on circulating Th17 cells and IL-23R protein and mRNA expression. Circulating Th17 cells were analysed in PBMCs. Representative flow cytometry contour plots showing (A) percentage of IL23R+CCR6+ Th17 cells within CD3+CD4+CD45RO+ cells in G (green frame) and A (red frame) donors. Isotype controls are shown. IL-23R expression was analyzed in purified total CD4+ T cells by flow cytometry and qRT-PCR. (B) mRNA expression level of IL-23R in total CD4+ T cells in G (green squares) or A (red squares) donors. (C) Representative flow cytometry contour plots showing percentage of IL23R+ cells within purified CD4+ T cells in G and A donors. Isotype controls are shown. (D) Percentage of total CD4+ T cells expressing IL-23R in G or A donors. (E) Representative flow cytometry histogram showing median fluorescence intensity (MFI) as measurement of IL-23R expression in a G (green line) and an A (red line) donor. Isotype control is shown (tinted grey) (F) CD4+IL-23R+ T cells MFI in G (green) or A (red) healthy individuals. (G) Representative flow cytometry contour plots showing percentage of CD45RO+IL17+ cells within CD3+CD4+cells determined for G and A donors by intracellular cytokine staining. Isotype controls are shown Each symbol in panels B, D and F corresponds to a value obtained from an individual and horizontal bars represent means. Unpaired t test was performed yielding P values >0.05 for all panels. (TIF) [file pone.0017160.s001.tif]

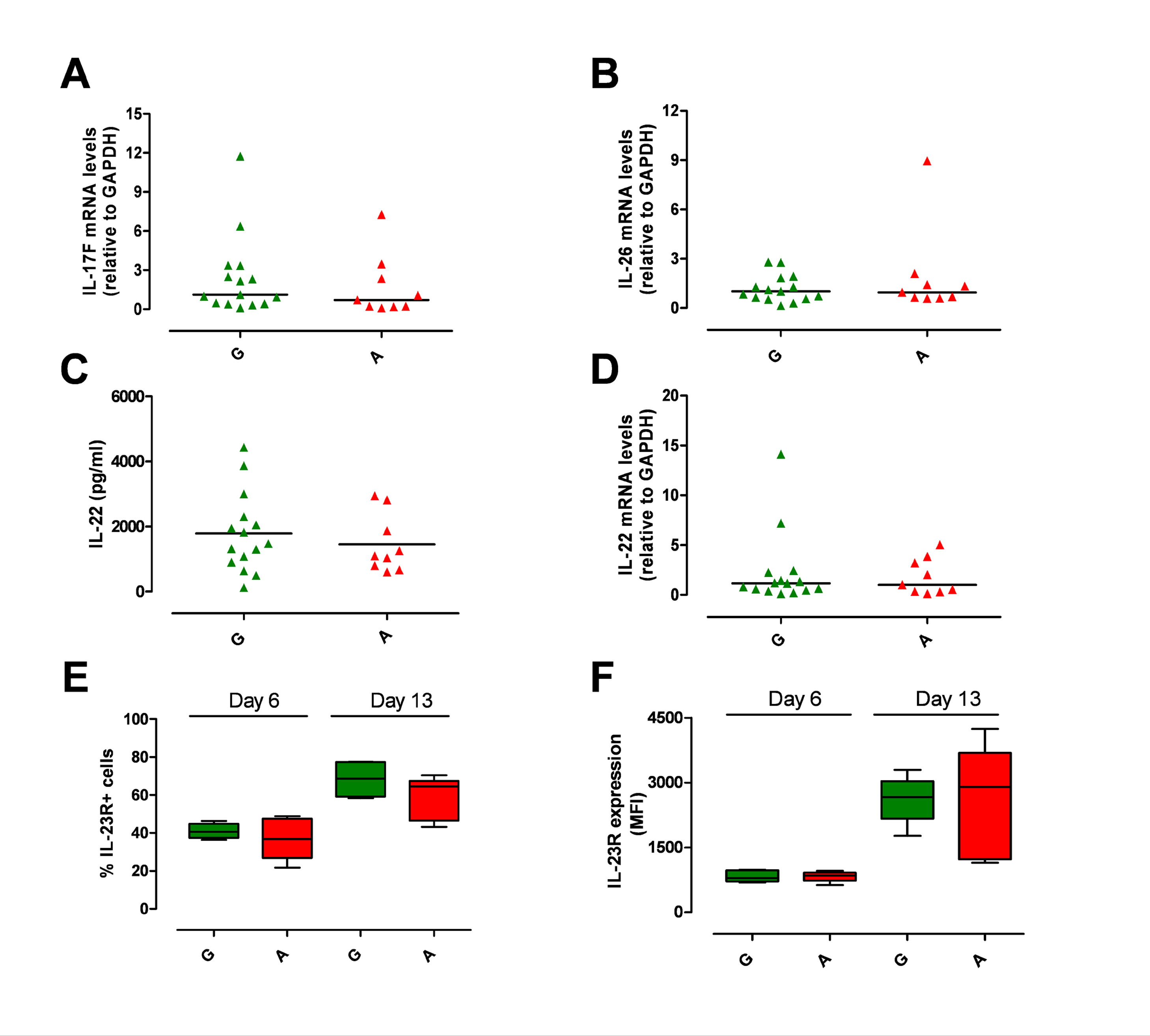

Supplement: Figure S2 — Effect of IL23R R381Q gene variant on Th17cell differentiation. IL-1β/IL-23 polarized Th17 cells were collected after 13d of culture, rested and then assayed for mRNA expression (on d14) or Th17 cytokine secretion (on d15). IL-17F (A) and IL-26 (B) mRNA and IL-22 protein secretion (C) and mRNA expression (D) in IL-1β/IL-23 polarized Th17 cells did not differ between G (green triangles) and A (red triangles) group. Time course analysis of IL-23R expression in IL-1β/IL-23 polarized Th17 cells was performed at d6 and 13 of culture by flow cytometry. Percentage of IL-23R+Th17 cells (E) and IL-23R MFI (F) did not differ between G (green boxes) and A (red boxes) group neither at d6 nor at d13. Box and whiskers of 5 donors per group are shown. Horizontal bars represent means (A) or medians (B, C, D, E, F). Unpaired t test (A) or Mann Whitney test (B, C, D, E, F) were performed yielding P values >0.05 for all panels. (TIF) [file pone.0017160.s002.tif]

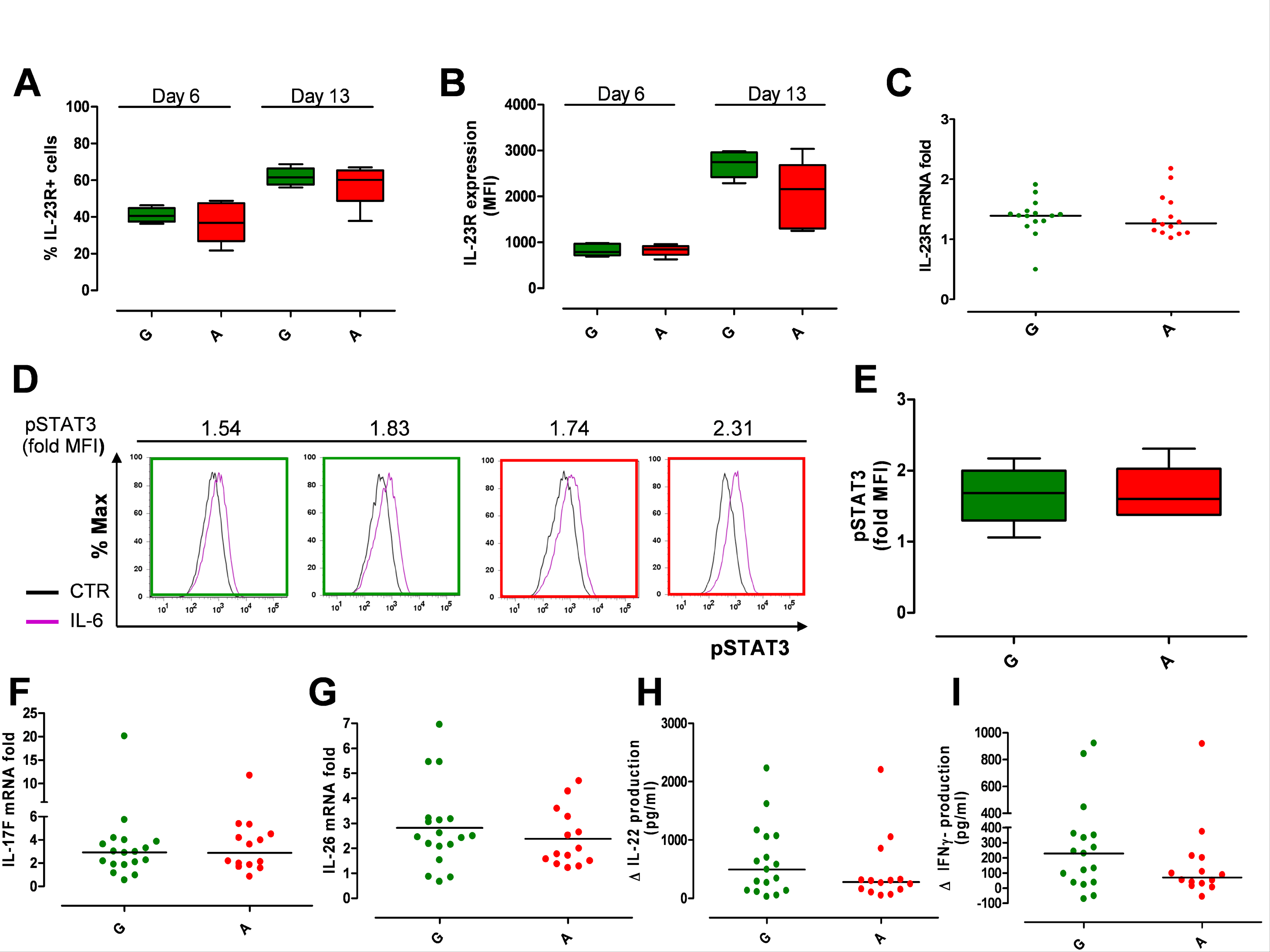

Supplement: Figure S3 — Effect of IL23R R381Q gene variant on IL-23R expression, IL-6-induced STAT-3 phosphorylation and production of pro-inflammatory cytokines. Time course analysis of IL-23R expression in IL-1β polarized Th17 cells was performed at d 6 and d13 of culture by flow cytometry. % of IL-23R+Th17 cells (A) and IL-23R MFI (B) did not differ between G (green boxes) and A (red boxes) group neither at d6 nor at d13. IL-1β-polarized Th17 cells were stimulated with IL-23 on day 13 of culture and IL-23R mRNA was measured on d14. IL-23R mRNA expression (C) in response to IL-23 stimulation (10 ng/ml) did not differ in IL-1β-polarized Th17 cells from A (red dots) versus G (green dots) group. mRNA were measured by qPCR and data are expressed as fold increase compared to un-stimulated cells. IL-1β-polarized Th17 cells were stimulated with IL-6 (100 ng/ml) for 15 min on d13 of culture and pSTAT-3 was measured by flow cytometry. (D) Representative flow cytometry histograms from two G (green frame) and two A (red frame) donors showing pSTAT-3 in response to IL-6 (pink line) as compared to unstimulated control cells (black line) and expressed as fold Median fluorescence Intensity (MFI). (E)Th17 cells from group G and group A did not differ in IL-6-induced pSTAT3. IL-1β-polarized Th17 cells were stimulated with IL-23 on d13 of culture and Th17 cytokine mRNA and proteins were measured on d14 or d15, respectively. IL-17F (F) and IL-26 (G) mRNA expression, as well as net IL-22 (H) and IFN-γ (I) production in response to IL-23 stimulation was not affected in IL-1β-polarized Th17 cells from A compared to G group. IL-17F and IL-26 mRNA were measured by qPCR and data are expressed as fold increase compared to unstimulated cells. For panels A, B and E box and whiskers of 4–5 donors per group are shown. In panels C, F-I each symbol corresponds to a value obtained from an individual. Horizontal bars represent medians (C, F, H, I) or means (G). Mann Whitney (C, F, H, I) or unpaired t test (G) was perform [file pone.0017160.s003.tif]
